# ‘The hospital and everyday life are two worlds’: Patients’ and healthcare professionals’ experiences and perspectives on collaboration in the kidney transplantation process

**DOI:** 10.1002/nop2.349

**Published:** 2019-08-10

**Authors:** Charlotte Nielsen, Hanne Agerskov, Claus Bistrup, Jane Clemensen

**Affiliations:** ^1^ Department of Nephrology Odense University Hospital Odense Denmark; ^2^ Department of Clinical Research University of Southern Denmark Odense Denmark; ^3^ HCA Research Odense University Hospital Odense Denmark; ^4^ Centre for Innovative Medical Technology Odense University Hospital Odense Denmark

**Keywords:** chronic disease, kidney transplantation, nephrology nursing, patient perspectives, qualitative research

## Abstract

**Aim:**

To explore patients’ and healthcare professionals’ experiences and perspectives on collaboration in the kidney transplantation process.

**Design:**

A qualitative study with a phenomenological‐hermeneutic approach.

**Method:**

Participant observation and interviews were conducted with 18 patients, together with a focus group with eight healthcare professionals from April 2016–January 2017. The data analysis was inspired by Ricoeur's theory.

**Results:**

While patients acknowledged that the healthcare professionals were experts, they also requested an everyday life approach to treatment and care, because both professional knowledge and everyday life experiences were needed to manage everyday life. A contrast between patients’ experiences and healthcare professionals’ knowledge was identified, and the empowerment approach could be a way to combine the different perspectives.

## 1 INTRODUCTION

This paper presents an explorative qualitative study of collaboration between patient and healthcare professional (HCP) in the kidney transplantation process. The study is part of a larger investigation, in three phases, which is concerned with developing new ways to structure and improve the transplantation process based on patients and HCPs’ experiences. The first phase is an identification of needs, through an exploration of experiences. Phase two is the design and development of a telehealth solution, to support the needs identified in the findings from phase one. Finally, in phase three, the telehealth solution is tested in clinical practice (Clemensen, Rothmann, Smith, Caffery, & Danbjorg, 2017). This paper reports from the first phase and includes patients’ experiences of the kidney transplantation process from acceptance for transplantation until four months post‐transplantation and the HCPs’ perspectives on the transplantation process.

In total, 554 individuals were enlisted for kidney transplantation in Denmark in 2018 and 236 patients received a kidney from either a living or a deceased donor (Scandiatransplant, 2019). Kidney transplantation implies survival benefit, higher quality of life, reduced medication, fewer restrictions for patients with end‐stage renal disease compared with dialysis and is the treatment of choice whenever feasible (Landreneau, Lee, & Landreneau, 2010; Oniscu, Brown, & Forsythe, 2005). Kidney transplantation, however, requires patients to manage lifelong self‐monitoring, medical adherence, careful hygiene and sufficient fluid intake to ensure successful transplantation outcomes. Patients must also be aware of side effects, such as increased risk of skin cancer and infections and in the long‐term increased morbidity (Wu et al., 2005).

## 2 BACKGROUND

Being a kidney recipient implies living with a chronic disease. As is the case for patients with other chronic diseases, it involves self‐management. This means taking responsibility for one's own care and treatment, by taking an active part in managing one's health condition and reacting to symptoms (Simmons, Wolever, Bechard, & Snyderman, 2014). It requires patients to have knowledge about their disease and confidence to manage the chronic conditions based on their knowledge. This kind of patient involvement in treatment and care is significant if self‐management is to be successful (Simmons et al., 2014).

A systematic review of literature exploring patients’ perspectives has identified the main challenges concerning self‐management in kidney transplant recipients (Jamieson et al., 2016). They found self‐management is facilitated by a motivation towards adherent behaviour, a feeling of control over one's health, respect for and indebtedness to the donor and gratitude towards the medical team. In contrast, lack of self‐management can occur because of insufficient guidance, forgetfulness, distress or other factors that challenge adherent behaviour. It could also be caused by the patients’ feelings that everyday life is being overshadowed by disease and by being a patient. Support for and improvement of patients’ self‐management is significant to a positive outcome in kidney transplantation (Jamieson et al., 2016). Another study investigated self‐management challenges and support needs experienced by kidney transplant recipients (Been‐Dahmen et al., 2018). Patients have to become experts in being a kidney recipient, which implies managing treatment, forming a long‐lasting relationship with HCPs, adjusting daily life activities, improving self‐image and dealing with social and emotional issues (Been‐Dahmen et al., 2018). To initiate self‐management, patients need a kind of support that is holistic. To date, the focus has been on medical challenges and has overlooked support for the emotional and social challenges. Furthermore, to initiate self‐management, the relationship between patient and HCP has to be built on trust (Been‐Dahmen et al., 2018).

Another study exploring kidney recipients and HCPs’ experiences finds that a need for a holistic approach to care is acknowledged by both parties to strengthen quality of care in kidney transplantation. By treating the aspects concerning the whole person and not just the disease, it improves quality of life and minimizes the burden on everyday life (Brett, Ertel, Grimshaw, & Knoll, 2018). There is a need to combine the basic clinical approach, with a focus on the patient–HCP relationship and the kind of aspects that is most important to patients (Brett et al., 2018). Other studies have also focused on the importance of a holistic and individually tailored approach to care and treatment in the kidney transplantation process to develop patients’ individual everyday competences (Urstad, Wahl, Andersen, Øyen, & Fagermoen, 2012; Wiederhold, Langer, & Landenberger, 2011).

In summary, these studies show that self‐management and the relationship between patient and HCP are significant to the clinical outcome of transplantation and patients’ everyday life. Self‐management can be supported by an individual approach that addresses the issues most important to patients. Collaboration between patient and HCP can provide knowledge of patient preferences and experiences that can support the individual approach and the patient–HCP relationship. Thus, it is significant that collaboration needs to be explored, as it is an element in improving self‐management and thereby improve treatment and care in the kidney transplantation process.

## 3 RESEARCH QUESTION

What are patients’ and healthcare professionals’ experiences and perspectives on collaboration in the kidney transplantation process?

## 4 DESIGN AND METHOD

The study was designed as a qualitative, explorative study with a phenomenological‐hermeneutic approach inspired by the French philosopher Ricoeur's thoughts on narrative and interpretation (Ricœur, 1976). Data on the patient perspective were collected through participant observations (Spradley, 1980) and semi‐structured interviews (Kvale & Brinkmann, 2014). The HCP perspective was discussed in a focus group (Kitzinger, 1995). The combination of the three methods provided the opportunity to gain unique, in‐depth knowledge (Malterud, 2001).

### 4.1 Setting

The study was conducted in a Danish kidney transplant centre. During the transplantation process, patients met HCPs at the outpatient clinic and underwent an evaluation programme consisting of several medical examinations and physical tests prior to acceptance for transplantation. The acceptance or rejection was finalized with a consultation with the nephrologist. During transplantation, the patients were admitted for approximately one week. Subsequently, the patients were discharged to close follow‐up at the outpatient clinic for consultations with the nephrologist and informal meetings with the nurses working at the clinic.

### 4.2 Participants

Inclusion was conducted using purposeful sampling to make sure that the participants could provide various aspects of how the kidney transplantation process was experienced (Malterud, 2001). The patients were included from different stages along the transplantation process, that is before, during and four months after transplantation. Inclusion criteria were as follows: wait‐listed patients or kidney recipients over the age of 18 who spoke Danish.

In total, 18 patients were included: six women and 12 men, with a mean age of 53 (Range: 33–73). Patients were included in participant observation or interviews. For participant observation, 12 accepted participation. Three pre‐transplant patients declined because they found it difficult to talk about their situation. For individual interviews, 11 participated. Due to lack of mental and physical resources, five wait‐listed patients and four patients from the early postoperative stage declined participation. Furthermore, one patient withdrew from interview four months after transplantation. Five patients participated in both participant observations and interviews. Patients’ characteristics are presented in Table 1.

**Table 1:** Patients’ characteristics—participant observation and interviews

Participant	Sex	Age (Years)	TX type	Participation	Stage
P1	Male	57	Living	Observation	Before
P2	Male	55	Living	Observation	Before
P3	Male	33	Living	Observation	Before
P4	Female	42	Deceased	Observation	Before
P5	Male	49	Deceased	Interview	Before
P6	Male	51	Deceased	Interview	Before
P7	Female	67	Deceased	Interview	Before
				Observation	During
P8	Male	42	Living	Observation	During
P9	Female	43	Living	Observation	During
				Interview	After
				Observation	After
P10	Male	68	Deceased	Observation	During
P11	Male	68	Living	Interview	During
P12	Male	61	Deceased	Interview	During
P13	Male	43	Living	Interview	During
P14	Female	39	Living	Interview	During
P15	Female	66	Deceased	Observation	After
				Interview	After
P16	Female	37	Living	Observation	After
				Interview	After
P17	Male	73	Deceased	Interview	After
P18	Male	57	Deceased	Observation	After

[Correction added on 8 October 2019, after first online publication: In Table 1, the text in parenthesis throughout the table have been removed from this current version.]

The HCPs who participated in the focus group were doctors and nurses who had various experiences working with kidney transplantation recipients. They all worked at the same transplant centre. The HCPs are presented in Table 2. Inclusion criteria were as follows: HCP with experience of working at the transplant centre. In total, eight HCPs were included: six women and two men, with a mean of 13 years of experience (Range: 2.5–22). None declined to participate.

**Table 2:** Focus group HCP participants’ characteristics

Participant	Sex	Experience (Years)
HCP 1	Female	4
HCP 2	Female	14
HCP 3	Male	22
HCP 4	Male	20
HCP 5	Female	11
HCP 6	Female	18
HCP 7	Female	2.5
HCP 8	Female	14

## 5 DATA COLLECTION

Data were collected by the first author from April 2016–January 2017. The first author is experienced in qualitative research and renal care; however, she is not involved in clinical work.

### 5.1 Participant observation

Participant observation was conducted with inspiration from Spradley's nine domains, which provided the structure and supported an open‐minded approach during the observations (Spradley, 1980). Field notes were written and transcribed immediately afterwards. Participant observation was performed by following three groups of four participants. The first four participants were followed during a three‐day, in‐hospital evaluation programme to be accepted for transplantation. Four participants were followed on the ward before and after the transplant operation and during the first outpatient consultations. Finally, four patients participated approximately four months after the kidney transplantation at the outpatient clinic. In total, 150 hr of participant observation was conducted. Field notes contained the researcher's description of the observations and short quotations from informal interviews.

### 5.2 Individual interviews

A semi‐structured interview guide with open‐ended questions was used during interviews. It gave the participants the opportunity to narrate about their experiences (Kvale & Brinkmann, 2014). Literature and data from the participant observations were used to develop the interview guide. Interviews were performed with three participants, allowing them to tell about their experiences of being on the waiting list for kidney transplantation. The interviews were planned to take place before their transplantation; however, two participants received their transplant before the interview. Post‐transplantation, four participants were interviewed. The interviews took place in their homes or at the hospital, approximately five weeks post‐transplantation. Finally, four participants were interviewed in their homes approximately four months post‐transplantation. The interviews were recorded and lasted 18–83 min.

### 5.3 Focus group

Interaction in focus groups is important. Thus, the aim was to encourage the participants to talk to each other rather than addressing the facilitator (Kitzinger, 1995). The session was planned thoroughly in relation to, for example, seating, refreshments, introduction and the moderator role. Presentations and illustrations were used before each discussion of two topics: ‘Patient involvement’ and ‘patients’ experiences of the healthcare system’. An open‐ended interview guide was developed to support group reflection and discussion with questions, such as, for example: ‘What are your reflections when patients express a need to be involved in their treatment and care? ’ (Kitzinger, 1995; Krueger & Casey, 2015). The first author acted as moderator and the participants were four doctors and four nurses. The last author participated as co‐moderator, observing non‐verbal communication and supplemented with elaborating and clarifying questions. The setting was a conference room at the hospital; however, it was not connected to the department. The focus group lasted two hours and was recorded.

The first author transcribed verbatim the field notes and recordings from interviews and the focus group. The entire research team, consisting of the authors, planned the research process and discussed the findings in the study. This researcher triangulation and the use of various methods enhanced the study validity (Malterud, 2001). Furthermore, the consolidated criteria for reporting qualitative research (COREQ guidelines) were followed in reporting the study (Tong, Sainsbury, & Craig, 2007).

### 5.4 Ethical considerations

In accordance with applicable ethical rules (Helsinki, 2018), the participants were informed orally and in writing about the study and written informed consent was obtained. The Danish Data Protection Agency approved the study, journal number: 15/48886. Approval from the National Committee on Health Research Ethics was not required by Danish law.

### 5.5 Data analysis

**Figure 1:**
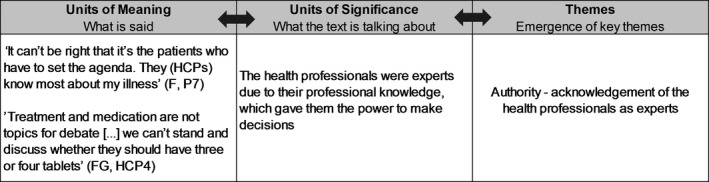
Example of the structural analysis

Text material from transcripts of field notes, interviews and the focus group were analysed as one coherent text. Inspired by Ricoeur's philosophy of interpretation, the analysis was conducted in a dialectic movement between three levels: Naïve reading, structural analysis and critical interpretation and discussion (Ricœur, 1976). The first impression of the text was gained in the naïve reading. During the structural analysis, themes emerged when the text was opened up in ‘units of meaning’ and ‘units of significance’ were identified in a movement from the participants’ quotations of ‘what is said’ to ‘what the text is talking about’. The themes were identified in an ongoing internal validation between the naïve reading, the ‘units of meaning’ and ‘units of significance’—as illustrated in Figure 1 (Pedersen, 1999/2005; Ricœur, 1976). The software programme NVivo 11 was used to systemize the analysis.

This represents the beginning of a movement from an individual to a general level, which will be further unfolded in the following presentation of the final critical interpretation and discussion of the emerged themes, with the help of theory and research literature. The themes is presented by illustrative quotations representing ‘what is said’ and will be followed by an interpretation of ‘what the text is taking about’ representing the meaning of the lived experience as a whole from across the entire data set (Dreyer & Pedersen, 2009; Pedersen, 1999/2005).

## 6 RESULTS

The naïve reading revealed that the patients and HCPs seemed to have various experiences of collaboration during the transplantation process. From both perspectives, the HCPs seemed to be acknowledged as experts. However, the relationship between patient and HCP also seemed to be influenced by opposing perspectives.

**Figure 2:**
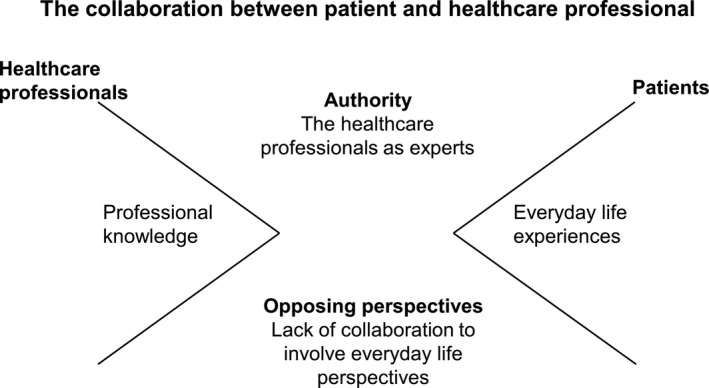
Illustration of the findings

The structural analysis led to two themes related to collaboration: ‘Authority – acknowledgement of the healthcare professionals as experts’ and ‘patients’ opposing perspectives in the relationship with the healthcare professionals’. A summary of the findings is illustrated in Figure 2. The themes will be interpreted in the following section. The quotations will be referred to as follows: (I) refers to interview, (F) refers to field note, (FG) refers to focus group, (P) refers to patient and (HCP) refers to healthcare professional.

### 6.1 Authority—acknowledgement of the healthcare professionals as experts

Both patients and HCPs perceived the HCP’s role as being that of an expert in relation to treatment. Expressed by a patient:

It can’t be right that it’s the patients who have to set the agenda. They [HCPs] know the most about my illness’. (F, P7)

And one HCP said:

They come to the hospital and it is us who decide, for example, you have to come at that time, you will have blood tests taken before that and you must take that medicine, etc. (FG, HCP3)

Reflections on the role of the HCPs in relation to knowledge and treatment showed how both parties acknowledged the HCPs’ responsibility and gave them control and power in their professional relationship. Being an expert in kidney transplantation implied responsibility for the treatment, the functioning of the kidney graft and the patient's health. It gave power to make decisions on behalf of the patients. As one HCP said:

Treatment and medication are not topics for debate […] we can’t stand and discuss whether they should have three or four tablets’. (FG, HCP4)

Healthcare professional had the responsibility for the medical treatment and the patients fully acknowledged that. They respected the HCPs as experts due to their professional knowledge and gave them power to make decisions regarding treatment. The HCPs’ authority as experts provided reassurance and confidence among the patients. The experience of the HCPs as experts depended not only on what was explicitly said, but also on the non‐verbal communication:

They [HCPs] know exactly when one starts getting better […] they are talented, they’ve seen it a million times before […] that knowledge makes me feel secure […] I really feel that if they have an attitude of calm and certainty, then I have it too’. (I, P14)

The HCPs could inspire confidence in the way they acted due to their experience and professional knowledge. It was significant for the patients because it provided comfort and made them feel safe.

Follow‐up was important to monitor the kidney functioning and was also seen as an opportunity to make sure that the patients were taking their medication as prescribed. There was an anticipation that HCPs needed to ‘keep an eye on’ the patients. The HCPs said:

The follow‐up sends a signal to the patients that, with this, it is important to keep a close eye on you (FG, HCP2) and …if they don't ask for new medication, it can lead to a doubt about whether they are taking it at all. (FG, HP3)

The HCPs’ role as experts involved the assumption of authority that they felt was needed in the collaboration with the patients. The HCPs had a lack of confidence in the patients’ ability to self‐manage, that is to comply with the responsibility that came with being a kidney recipient. HCPs experienced that the patients needed their control and support to accommodate the obligations as a kidney recipient. This established a paternalistic approach to the patients and created an asymmetric relationship between patients and HCPs. The HCPs described the HCP–patient relationship experienced by the patients as a metaphor—that is ‘being a family’. One HCP expressed:

But they are willing to drive a long way [citing the patients]. It’s here that I was transplanted, we’ll just see and when a year has passed, we want to keep coming here. They almost feel like we are family. (FG, HCP 4+3)

The HCPs experienced patients had confidence in them. The patients felt a strong connection with the HCPs and were dependent on them as experts. This further substantiated the HCPs as the authority in the relationship.

The patients acknowledge the role of the HCP as an expert. In that way, both groups acknowledged the asymmetry of the collaboration, in that the HCP held the power, by keeping the patients safe and confident. This could lead to a contradiction, because the patients had perspectives in the collaboration, other than acknowledging the HCP’s role as expert.

### 6.2 Patients’ opposing perspectives in the relationship with the healthcare professionals

Being a patient felt like being subject to others, because it involved having to adapt to the healthcare system by conforming to norms, rules and professional knowledge:

…Then an HCP comes in around 6‐7 o'clock. They are going to take a blood test, so you have to get up, then you have to get weighed and then you have your temperature taken and it goes on in that rhythm, as is the workflow in a hospital. (P12, I)

It was a transformation from being independent and performing self‐management at home into a state where one had to rely on the HCPs’ professional knowledge and assessment. One patient expressed:

It is really unpleasant to be overruled that others suddenly know best about my body, I have had a hard time with that. (I, P14)

The situation as kidney recipient was new and unknown to the patients, and they could not recognize their body reactions and symptoms, so they found confidence in the HCPs’ professional knowledge, as a way to understand the situation. Though, it was difficult for the patients to be dependent on the HCPs. The patients relied on the HCPs and focused on meeting the HCPs’ expectations regarding patient compliancy and adherence.

In contrast to the HCPs’ professional knowledge were the patients’ everyday life experiences about how it was to live as a kidney recipient. The patients felt that the HCPs’ biomedical focus was a way for them to interpret the patient's condition and well‐being:

HCPs look a lot at blood tests and if they are okay, then there isn’t really anything […], if you have anything to say to the contrary, it doesn't matter because the blood tests say something different. (I, P9)

The patients felt the biomedical parameters offered a limited perspective, because they did not necessarily reflect how the patients experienced their well‐being in everyday life. There could be challenges at home in family life, side effects from medication, mental limitations like tiredness or other issues at stake for the patients—despite the fact that the kidney graft was functioning well. It would require collaboration by asking questions and involvement of the patients, for the HCPs to get knowledge of how the patients experienced their well‐being.

Preparation, by way of seeking knowledge and asking questions, provided an opportunity for the patients to facilitate collaboration. Also, to gain a better understanding of the treatment and how, in dialogue with the HCPs, to manage the situation as a kidney recipient:

I am getting prepared, so that I can discuss things with the doctor, so that I don’t just come in and tell him how it is going, but I can question something and say should we not soon reduce it [reduce medication dosage] and should we not do this or that. (I, P13)

Patients made preparations for the meeting with the HCP to achieve the role of collaborative partner in treatment and decision‐making. The patients all strived for collaboration to achieve involvement and self‐management. Some tried to have control over the timing of appointments at the hospital with a view to disrupting everyday life as little as possible. Others prepared for a dialogue about treatment so that their everyday life would be affected by the fewest possible side effects. The patients experienced that their perspectives were welcomed by the HCP.

The HCPs acknowledged the significance of collaborating with the patients. One HCP said:

It is important that patients know their medication, it is part of being a patient, managing their medicine, so they can just as well do it while they are in hospital. (FG, HCP1)

Yet, it could also be challenging for them to involve the patients instead of rely on their own professional assessment:

It is not like a menu, where the patient says, no, next time I would like a telephone consultation […] It is an individual assessment [by the HCP]. (FG, HCP3)

The HCPs were positive towards self‐management and collaboration and expressed that supporting patients, in developing everyday life competences, was significant; however, it was a challenge to involve patients, because they perceived it as handing over power to the patients. It was a contrast to the HCPs’ perception of their role as an expert and the asymmetric patient–HCP relationship.

There was a lack of coherence between being at the hospital and everyday life at home. At the hospital, decisions about treatment and care often were made by the HCPs solely:

The hospital and everyday life are two worlds; in the hospital you are told what to do and you are constantly waiting for someone to say what’s going to happen. In everyday life you have to make decisions yourself. (F, P1)

A contrast was seen between hospital and everyday life. At the hospital, self‐management was not a focus and the HCP held the initiative in treatment and care. The HCP often conducted observations and task in relation to treatment and care without any explanations to the patients. This did not prepare the patients for their responsibility at home, and it could be a challenge to transfer professional knowledge and recommendations into actions to take care of the kidney graft or react to possible symptoms related to the kidney graft in everyday life.

Patients strived towards collaboration with the HCP. Collaboration was found significant to provide knowledge of patients’ experiences, patient involvement and preparation for everyday life at home. Thus, there were opposing perspectives represented of both the HCP as an expert and a collaborative partner.

## 7 DISCUSSION

In this study, the HCPs’ role was established as that of the expert, due to their professional knowledge and experience in the kidney transplantation process. The patients acknowledged and requested their knowledge and strived to remain compliant with the HCPs’ professional recommendations. However, it was significant that everyday life experiences were included together with biomedical knowledge in decisions about treatment and care—such as issues regarding family, work and limitations. This could challenge the HCPs’ biomedical approach and expert role in the collaboration between patient and HCP.

Other studies describe patients’ perception of HCPs as experts, similar to the finding in our study. Patients experience to be subject to the HCPs’ professional knowledge during the entire transplantation process (Low, Crawford, Manias, & Williams, 2017; Schmid‐Mohler, Schäfer‐Keller, Frei, Fehr, & Spirig, 2014; Tong et al., 2015). Wait‐listed patients are dependent on the HCPs’ conclusion of evaluation to be accepted for kidney transplantation and to stay on the waiting list (Tong et al., 2015). Post‐transplantation patients experience stressors caused by adapting to a new chronic condition of being a kidney recipient. Patients have to rely on the HCPs’ recommendations and support. In one study, a patient continued taking his medication despite several side effects and found confidence in the promise of adjustment to a lower dose over time (Low et al., 2017). In another study, patients experienced a need for HCP support, because going through a kidney transplantation had led to change and instability in everyday life (Schmid‐Mohler et al., 2014).

In the current study, we found that collaboration between patient and HCP was needed to provide knowledge of patients’ everyday life experiences. The significance of everyday life experiences is found in other studies of kidney recipients (Been‐Dahmen et al., 2018; Brett et al., 2018; Urstad et al., 2012; Wiederhold et al., 2011). Going through kidney transplantation was revealed to be challenging for the patients, and they needed support and education. It was important that the approach taken was individual and the education was personally tailored, by way of the HCPs accessing the patients’ individual knowledge and education needs. The patients were looking for an approach, addressing not only the disease but also emotional and social challenges. This developed everyday life competences and self‐management with minimal burden on the patients’ lives (Been‐Dahmen et al., 2018; Brett et al., 2018; Urstad et al., 2012; Wiederhold et al., 2011). This shows everyday life experiences, as a significant complement to the professional knowledge that could add an individualized angle to treatment and care and thereby connect professional knowledge to the challenges in patients’ everyday life. This will develop everyday life competences and support patients’ ability to self‐management at home. Both professional knowledge and everyday life experiences are needed to manage life as a kidney recipient, and it is collaboration between patient and HCP that can combine the two perspectives. However, we found the collaboration was challenged by the HCP’s role as an expert.

Interactional nursing practice theory, developed by Scheel, Pedersen, and Rosenkrands (2008), contributes to a deeper understanding of the collaboration and perspectives of patient and HCP. It provides a philosophical understanding of the relationship as a dynamic field that contains natural, human and social sciences. Reason and emotion are integrated in the practice theory, which is based on Habermas’ theory and definition of three types of reason, with corresponding modes: cognitive‐instrumental, aesthetic‐expressive and moral‐practical. According to Scheel et al. (2008), all three modes are complementary and necessary in interactional nursing. In our study, the HCPs’ role was established as an authority due to their professional knowledge and biomedical approach to patients. According to Scheel et al. (2008), this can be explained as a *cognitive‐instrumental* mode of action. It is a result‐oriented approach that focuses on the patient's bodily needs and instrumental and technical activities, corresponding with natural science (Scheel et al., 2008). The cognitive‐instrumental action is important and necessary for HCPs to provide treatment and care based on professional and biomedical knowledge. An asymmetrical relationship between patient and HCP was found in the current study due to the HCPs’ professional knowledge, but their power was used with moral responsibility in favour of the patients. According to Scheel et al. (2008), ethics are always present in relations between people, a condition from which, as human beings, we cannot free ourselves. It is important that we act with moral responsibility for one another. This can be explained as the *moral‐practical* mode of action (Scheel et al., 2008). Our finding of opposing perspectives in the collaboration between patients and HCPs arose when the patients requested knowledge and guidance that could be adapted to their everyday lives, in contrast to a general biomedical professional knowledge. This can be described as an example of Scheel et al.’s (2008) a*esthetic‐expressive* mode of action, where the focus is on reaching a mutual understanding in a dialogue between patient and HCP. By combining the professional knowledge with the patients’ experience, regarding aspects such as family, work and limitations, a person‐specific mutual understanding can be reached. This thereby supports the patients to handle their situations in everyday life (Scheel et al., 2008).

The contradictory findings of the importance of the HCPs’ professional knowledge coupled with patients’ expectations of a more individually adapted knowledge of everyday life can be explained as an emerging contrast between system and lifeworld (Scheel et al., 2008). Habermas describes the society as divided into a system and a lifeworld, each regulated by different actions. The system is regulated by money and power and human action is impersonal. In contrast, the lifeworld is regulated by understanding each other and communication, and the intention of human action is to achieve a mutual understanding (Scheel et al., 2008). The contrast in the relationship between patient and HCP arises because the treatment and care take place in the system, but are connected to the patient's lifeworld. To accommodate the contrast, Scheel et al. (2008) argue that HCPs must strengthen the values of the lifeworld in the system where the relationship with patients unfolds. It is important not to focus solely on cognitive‐instrumental knowledge. Otherwise, the system will colonize the lifeworld (Scheel et al., 2008). The contrast illustrates a difference between the HCPs’ professional knowledge and the patients’ individual needs and everyday life experiences.

Improvement of collaboration between patients and HCPs could be an approach to facilitate empowerment, because collaboration increases the focus on the patients’ perspectives. The empowerment approach, in the study by Anderson and Funnell (2010), is developed to facilitate self‐directed behaviour change in diabetes care. Similar to patients with diabetes, kidney recipients undergo behaviour changes in regard to significant aspects of everyday life, such as self‐monitoring, observations of symptoms and compliance with diet and fluid intake recommendations. Empowerment implies acknowledging that patients make decisions and have control of their daily care. The HCPs’ role is to collaborate and support patients to make informed choices, with knowledge of the consequences (Anderson & Funnell, 2010). This challenge the findings in our study related to the HCPs’ expert role. However, it meets the patients’ experiences of lack of coherence between hospital and everyday life and support patients’ self‐management in everyday life with their chronic condition as a kidney recipient. Two distinct kinds of expertise are equally present in the empowerment approach: that is, the HCPs’ expertise regarding what is best in relation to the disease and the patients’ expertise regarding what is best in relation to their lives, such as priorities, concerns, values and resources. Both kinds of expertise are necessary to plan treatment and care in consideration to both disease and everyday life (Anderson & Funnell, 2010). Thus, the professional knowledge addresses the everyday life experience in the empowerment approach, which we have identified were significant for patients in the kidney transplantation process. This acknowledges the significance of the professional knowledge and everyday life experiences to be used complementarily.

## 8 STRENGTHS AND LIMITATIONS

The study is a single‐centre study, which calls into question its transferability. However, our findings of patients’ experiences seem to be similar to other studies of the kidney transplantation process. Combined with the HCPs’ perspective, a new understanding of collaboration in the kidney transplantation process was achieved. The discussion involving interactional nursing practice theory (Scheel et al., 2008) and the concept of empowerment (Anderson & Funnell, 2010) bring new insights into the patient–HCP relationship, which can be related to nursing practice in general.

## 9 CONCLUSION

Two varying perspectives are present in the collaboration between patient and HCP in the kidney transplantation process, and a contrast is identified between professional knowledge and everyday life experiences. However, the patients and HCPs agree on the HCPs’ role as an expert. The contrast emerges between system and lifeworld. The patients experience a lack of involvement of their everyday life perspective; thus, it will be essential to strengthen the values of the lifeworld in the relationship between patient and HCP. The empowerment approach could be a way to strengthen the values of the lifeworld, because, in this approach, the value of everyday life experiences is equated with those of professional knowledge. Thus, the study identifies a need for a new approach in clinical practice to involve patients’ experiences in treatment and care, facilitated by collaboration, to support patients’ everyday lives during the kidney transplantation process.

## CONFLICT OF INTEREST

No conflicts of interest have been declared by the authors.
